# The link between personality dimensions, impulsivity, decision and coping style, and suicide attempts in affective patients.

**DOI:** 10.1192/j.eurpsy.2024.517

**Published:** 2024-08-27

**Authors:** J. M. Pawlak, K. Bilska, M. Skibińska, B. Narożna, P. Zakowicz, A. Rajewska-Rager, P. Kapelski, M. Dmitrzak-Węglarz

**Affiliations:** ^1^Departnet of Psychiatric Genetics; ^2^Department of Pneumonology, Pediatric Allergology and Clinical Immunology, Poznan University of Medical Sciences, Poznan, Poland

## Abstract

**Introduction:**

Introduction: Affective patients, especially depressive, have an increased risk of suicidal behavior. Identifying individuals at increased risk remains a challenge. Among the correlates that may be crucial, the impact of personality is emphasized. Attention is paid to impulsivity, measured by subjective or objective tests.

**Objectives:**

Objectives: Comparative analyses were carried out to capture the differences and relationship between personality dimensions, impulsivity, and the decision-making style and coping with stress strategies in suicide attempters and non-attempters in the course of an affective disorder.

**Methods:**

Methods: Data were obtained from 276 individuals diagnosed with unipolar and bipolar affective disorder, both sexes. The study group was disaggregated into a subgroup of patients with (N=95) and without (N=181) suicide attempts in an individual’s history. The Temperament and Character Inventory (TCI) was used to assess personality dimensions. The Barratt Impulsiveness Scale version 11 (BIS-11) was used to measure impulsivity subjectively, and the Simple Reaction Time (SRT) test and the Continuous Performance Test (CPT) were objective assessment methods. The Coping Orientation to Problems Experienced (COPE) and Iowa Gambling Task (IGT) were applied to investigate coping and decision-making styles. Statistical analyses were performed in Statistica 13.3 StatSoft, Krakow, Poland.

**Results:**

Results: In TCI, significant differences between suicide attempters and non-attempters concerned the following dimensions: harm avoidance (HA) (p=<0.0000), self-directedness (SD) (p=0.0001), and cooperativeness (C) (p=0.0186). In the CPT test, significant differences concerned correctly responded trials (p=0.0179) and Bias response (p=0.0230). In IGT, significant differences occurred in IGT block1_sum (p=0.0496) only (Table 1). We did not observe any significant differences in other tests applied. In the Spearman rank correlation analysis in the group of suicide attempters, the following correlations (p>0.05) with at least moderate strength rs>0.4 were revealed: Novelty seeking (NS), SD, and C correlated with several CPT parameters; Persistence (P) correlated with SRT variables; NS, HA and SD with BIS-11 variables.

**Conclusions:**

Conclusions: Objective computerized tests (SRT; CPT; IGT) did not differentiate suicide attempters and non-attempters more clearly than self-reporting personality inventory TCI. Personality traits correlated with SRT and CPT variables. BIS-11 and COPE parameters did not enable to distinguish suicide attempters and non-attempters in the investigated group. This suggests that tests used complement each other, and using a single tool may be insufficient to indicate patients at increased risk of suicidal behavior.

**Disclosure of Interest:**

None Declared

